# Central CRF neurons are not created equal: phenotypic differences in CRF-containing neurons of the rat paraventricular hypothalamus and the bed nucleus of the stria terminalis

**DOI:** 10.3389/fnins.2013.00156

**Published:** 2013-08-30

**Authors:** Joanna Dabrowska, Rimi Hazra, Ji-Dong Guo, Sarah DeWitt, Donald G. Rainnie

**Affiliations:** Division of Behavioral Neuroscience and Psychiatric Disorders, Department of Psychiatry and Behavioral Sciences, Yerkes National Primate Research Center, Emory UniversityAtlanta, GA, USA

**Keywords:** CRF, BNST, PVN, Oxytocin, VGLUT2, GAD67, vasopressin

## Abstract

Corticotrophin-releasing factor (CRF) plays a key role in initiating many of the endocrine, autonomic, and behavioral responses to stress. CRF-containing neurons of the paraventricular nucleus of the hypothalamus (PVN) are classically involved in regulating endocrine function through activation of the stress axis. However, CRF is also thought to play a critical role in mediating anxiety-like responses to environmental stressors, and dysfunction of the CRF system in extra-hypothalamic brain regions, like the bed nucleus of stria terminalis (BNST), has been linked to the etiology of many psychiatric disorders including anxiety and depression. Thus, although CRF neurons of the PVN and BNST share a common neuropeptide phenotype, they may represent two functionally diverse neuronal populations. Here, we employed dual-immunofluorescence, single-cell RT-PCR, and electrophysiological techniques to further examine this question and report that CRF neurons of the PVN and BNST are fundamentally different such that PVN CRF neurons are glutamatergic, whereas BNST CRF neurons are GABAergic. Moreover, these two neuronal populations can be further distinguished based on their electrophysiological properties, their co-expression of peptide neurotransmitters such as oxytocin and arginine-vasopressin, and their cognate receptors. Our results suggest that CRF neurons in the PVN and the BNST would not only differ in their response to local neurotransmitter release, but also in their action on downstream target structures.

## Introduction

Corticotrophin releasing factor (CRF) is a neuropeptide that is essential for coordinating the adaptive response of an organism to stressful situations (Vale et al., 1981). CRF is produced predominantly by neurons in the parvocellular division of the paraventricular nucleus of the hypothalamus (PVN) (Swanson et al., 1983; Herman et al., 2003). However, CRF is also produced by neurons in extra-hypothalamic limbic structures like the extended amygdala, which includes the bed nucleus of the stria terminalis (BNST) and the central nucleus of the amygdala (CeA), as well as in hindbrain structures like the locus coeruleus and dorsal Raphé nuclei (Cummings et al., 1983; Curtis and Valentino, 1994; Snyder et al., 2012). CRF that is synthesized and released by PVN neurons plays a major role in regulating activity of the hypothalamic-pituitary-adrenal (HPA) axis and triggers the classic endocrine stress response (Vale et al., 1981; Rivier and Vale, 1983), whereas outside the HPA axis CRF acts not as a hormone, but as a modulator of synaptic transmission at pre- and postsynaptic sites within specific central neuronal circuits (Lowry and Moore, 2006; Orozco-Cabal et al., 2006). In the BNST, the highest concentration of CRF neurons are found in the oval and fusiform nuclei (Cummings et al., 1983; Morin et al., 1999), which is also rich in CRF fibers and terminals, many of which originate from the CeA (Cummings et al., 1983; Sakanaka et al., 1986; Morin et al., 1999). Growing evidence suggests that CRF neurons of the anterolateral cell group of the BNST, BNST_ALG_, play a major role in the affective response to stressors (Lee and Davis, 1997; Liang et al., 2001; Nijsen et al., 2001; Ciccocioppo et al., 2003; Sahuque et al., 2006; Dabrowska et al., 2013), and that dysfunction of this CRF system contributes to the etiology of several psychiatric disorders, including depression (Crestani et al., 2010), anxiety-disorders (Walker et al., 2009), as well as addiction (Koob, 2010).

However, despite sharing a common neuropeptide phenotype, central CRF neurons should not be regarded as a homogeneous cell population. For example, the Raphé nuclei contain a population of CRF neurons that co-localize serotonin (5-HT) (Valentino et al., 2010), whereas locus coeruleus CRF neurons co-localize noradrenalin (NA) (Valentino et al., 1983, 2010). Hence, target structures that receive input from CRF neurons in these two midbrain nuclei would be predicted to have a markedly different response to afferent input. Consistent with this premise, evidence from *in situ* hybridization and immunohistochemical studies suggest that PVN CRF neurons could have a glutamatergic phenotype (Ziegler et al., 2002; Lin et al., 2003; Hrabovszky et al., 2005; Hrabovszky and Liposits, 2008), whereas BNST CRF neurons could be GABAergic (Sun and Cassell, 1993; Bowers et al., 1998; Day et al., 1999; Pompolo et al., 2002). This raises the intriguing possibility that either CRF neurons in these two stress-reactive structures have a phenotype that is independent of the surrounding structure or that they co-express two functionally opposing neurotransmitters. Support for the latter premise comes from the observation that stress responsive neurons in the BNST, but not the PVN, are primarily GABAergic (Herman et al., 1996; Day et al., 1999; Bali et al., 2005; Janitzky et al., 2009). However, no study to date has systematically examined the neurochemical phenotype of CRF neurons in these two regions at the single cell level.

As noted above, the PVN and BNST also contain subpopulations of neurons that express a wide variety of neuropeptides including, but not limited to, oxytocin (OT), arginine-vasopressin (AVP), neurotensin (NT), and enkephalin (ENK) (Sawchenko et al., 1984a,b; Lightman, 1993), some of which are co-expressed in the same cell populations. For example, although AVP is primarily produced by magnocellular PVN neurons, under stress conditions parvocellular CRF neurons in the PVN also co-synthesize AVP (Sawchenko et al., 1984a; Arima et al., 2001), suggesting that the neurochemical phenotype of these neurons can be dynamically regulated in response to environmental demands. Interestingly, CRF and OT are also reported to be co-expressed in a subset of PVN neurons (Sawchenko et al., 1984b), and our recent single-cell RT-PCR study confirmed that some magnocellular OT neurons in the PVN also co-express mRNA transcripts for CRF (Dabrowska et al., 2011). Together these data suggest that stress responsive CRF neurons may have a phenotype that is both region-specific and dynamically regulated in response to environmental stimuli.

Finally, we recently reported that a reciprocal relationship exists between CRF neurons of the BNST_ALG_ and OT neurons of the PVN (Dabrowska et al., 2011). Hence, magnocellular OT neurons in the PVN express high levels of type 2 CRF receptors (CRFR2), whereas BNST_ALG_ CRF neurons express high levels of OT receptor mRNA (OTR), suggesting that OT neurons of the PVN might directly regulate the excitability of the CRF neurons in the BNST_ALG_ and *vice versa*. However, OT is also released locally in the PVN (Neumann, 2007), raising the possibility that CRF neurons in the PVN may also be regulated by local OT release. Indeed, we have shown that magnocellular CRF neurons in the PVN express OTR mRNA (Dabrowska et al., 2011), however, nothing is known about OTR expression in the parvocellular CRF cell population. Furthermore, although multiple neuronal subtypes have been characterized in the rat PVN (Tasker and Dudek, 1991) and BNST (Hammack et al., 2007; Hazra et al., 2011) based on their electrophysiological properties, little is known about the properties of CRF expressing neurons due to the lack of a cell-type specific marker for these neurons. To address these knowledge gaps, we have used a combination of dual-immunofluorescence and whole-cell patch clamp recording in conjunction with single-cell RT-PCR to probe rat CRF neurons in both regions for their expression of GABAergic (glutamic acid decarboxylase, GAD67) and glutamatergic (vesicular glutamate transporter, VGLUT) markers, as well as for other neuropeptides (OT, AVP) and their cognate receptors (OTR, V1AR, V1BR).

## Methods

### Animal subjects

All experiments were performed on brain tissue obtained from adult (60 days old), male, Sprague-Dawley rats (Charles River Laboratories, Wilmington, MA). Animals were housed 4 animals per cage and were maintained on a 12:12-h light–dark cycle with *ad libitum* access to food and water. For experiments that required stereotaxic surgery and colchicine injections, rats were anaesthetized with an IP injection of a mixture of dexdormitor (0.16 mg/kg; Pfizer Animal Health, New York, NY, USA) and ketamine hydrochloride (48 mg/kg; Butler-Schein Animal Health, Dublin, OH, USA). All procedures used were approved by the Institutional Animal Care and Use Committees (IACUC) of Emory University, and were in compliance with National Institutes of Health (NIH) guidelines for the care and use of laboratory animals.

### Tissue processing for immunofluorescence

To facilitate immunohistochemical analysis, 6 rats received a 2 μ l intracerebroventricular (icv) colchicine (Sigma Aldrich, St. Louis, MO, USA, 60 μ g/μl) infusion 48 h prior to transcardial perfusion to maximize CRF peptide content in neuronal cell bodies, as described previously (Dabrowska et al., 2011). Subsequently, all rats were transcardially perfused with 4% paraformaldehyde and subsequent fixation procedure was performed as described elsewhere (Dabrowska and Rainnie, 2010).

### Dual-immunofluorescence experiments

The specificity of the antibodies used in this study has been confirmed and described previously (Martin et al., 2010; Dabrowska et al., 2011). Dual-immunofluorescence experiments were performed using the following primary antibodies: rabbit polyclonal anti-CRF antibody (1:250, ab11133, Abcam, Cambridge, MA), mouse monoclonal anti-GAD67 (1:500, MAB5406, Chemicon-Millipore, Billerica, MA), mouse monoclonal anti-VGLUT2 antibody (1:1000, clone N29/29, 75-067, UC Davis/NIH NeuroMab Facility, Davis, CA, USA, Antibodies Incorporated) and using protocols that have been described previously (Dabrowska et al., 2011).

To examine the co-localization of CRF with GAD67 and VGLUT2, we performed dual-immunofluorescence experiments on free-floating serial sections of the rat BNST_ALG_ and PVN as described before (Dabrowska et al., 2011). Here, representative sections were taken from Bregma +0.22 to −0.4 mm for the BNST_ALG_, and from −1.6 to −2.12 mm for PVN. The sections were rinsed 3× (10 min each) in phosphate buffer saline (PBS), permeabilized with 0.5% Triton-X 100 in PBS, and incubated for 48 h at 4°C with the following primary antibodies pairs diluted in 0.5% Triton-X/PBS solution: CRF/GAD67 and CRF/VGLUT2. Sections were rinsed 3× (10 min each) in PBS and then incubated at room temperature for 2 h with Alexa-Fluor secondary antibodies specific for the primary antibody host: namely Alexa-Fluor 488 goat anti-mouse IgG and Alexa-Fluor 568 goat anti-rabbit IgG (1:500, Invitrogen, Carlsbad, CA, USA). Following incubation with secondary antibodies, sections were rinsed 3× in PBS and 1× in 0.05 M phosphate buffer (PB), mounted on gelatin-coated glass slides and coverslipped using mounting medium consisting of 12% mowiol (Sigma Aldrich, St. Louis, MO, USA) and 30% glycerol. Confocal spinning disk laser microscopy was used to analyze dual-immunofluorescence patterns and to obtain high-resolution photomicrographs using an Orca R2 cooled CCD camera (Hamamatsu, Bridgewater, NJ, USA) mounted on a Leica DM5500B microscope (Leica Mircosystems, Bannockburn, IL) equipped with a CSU10B Spinning Disk (Yokagawa Electronic Corporation, Tokyo, Japan). Analysis and semi-quantitative analysis of dual-labeled neurons in the PVN and the BNST_ALG_ was performed with Simple PCI 6.6 software (Hamamatsu Corporation, Sewickley, PA, USA). For each pair of primary antibodies, immunoreactive neurons from representative sections containing either the BNST_ALG_, or the PVN, (from three animals, thirty sections total) were counted and analyzed for dual-labeling. Co-localization was determined by capturing confocal Z-stack images and counting the number of neurons co-localizing the two markers (VGLUT2/CRF or GAD67/CRF) in all focal plans. Cell counts were then expressed as a percentage of the total number of neurons expressing CRF.

### Single-cell RT-PCR

Single cell RT-PCR (scRT-PCR) protocols and the procedures used to determine mRNA transcript expression in single cells of the PVN and BNST_ALG_ have been described in detail elsewhere (Dabrowska et al., 2011; Hazra et al., 2011). Briefly, following recording of the electrophysiological properties of neurons in each area, the cytoplasm was aspirated from recorded neurons into the patch recording pipette and then expelled into a microcentrifuge tube containing a reverse transcription (RT) cocktail (Applied Biosystems, Foster City, California). The RT product was then amplified in triplicate and screened for 18S rRNA expression. Only those cells positive for 18S rRNA were subjected to amplification with primers. Oligonucleotide primers used in the current study have been described elsewhere (Dabrowska et al., 2011; Hazra et al., 2011), with the exception of V1AR and V1BR, as well as VGLUT1 and VGLUT3. The primers used for V1 receptors were as follows: 5′-CGACACAGCAAGGGTGACAAGG-3′ and 5′-AGGAAGCCAG CAACGCCG-3′ (accession number NM_053019.2, 265 bp) for V1AR, and 5′-AGCATCAGTACCATCTCCAGG-3′ and 5′-TGGTCTCCATAGTGGCTTCC-3′ (NM_017205.2, 463 bp) for V1BR. The primers used for VGLUT were as follows: 5′-ACCCATCGGAGGCCAGATCG-3′ and 5′-GCCACTCCTCCCGC GTCTTGTGC-3′ (NM_053859, 416 bp) for VGLUT1 and 5′-GGAATCATTGACC AAGATGAGTTAGCTGA-3′and 5′-TTT AGGTGTTTCTGAGAAGT CTCCTTCGG-3′ (AY117026, 200 bp) for VGLUT3.

### Controls for the scRT-PCR

PCR conditions were optimized using total RNA isolated from rat BNST_ALG_ so that a PCR product could be detected from (250 pg−1 ng) of total RNA without contamination caused by non-specific amplification. For each PCR amplification, sterile water was used instead of cDNA as a control for contaminating artifacts. A second negative control was performed in which the RT enzyme was excluded from the initial reaction mixture for each amplification reaction. All of the control tests gave negative results throughout the study. In addition, all of the primers used in this study were intron-spanning to exclude amplification of genomic DNA. Moreover, the cell nucleus was never harvested while isolating mRNA from single cells further reducing the possibility of contamination by genomic DNA.

### *In vitro* whole cell patch-clamp recording of visually identified neurons in the PVN and the BNST_ALG_

*In vitro* patch-clamp recordings were performed as previously described (Guo and Rainnie, 2010; Hazra et al., 2011). In brief, rat were anesthetized using isoflurane and the brains rapidly removed and placed into ice-cold kynurenic-based artificial cerebrospinal fluid (ACSF_KA_), which contained (in mM): NaCl (130), KCl (3.5), KH_2_PO_4_ (1.1), MgCl_2_ (6.0), CaCl_2_ (1.0), NaHCO_3_ (30), glucose (10), and kynurenic acid (2). Subsequently, 350 μm slices containing the BNST_ALG_ or PVN were obtained using a Leica VTS-1000 vibrating microtome (Leica Microsystems, Bannockburn, IL). ACSF_KA_ was used to minimize any potential excitotoxicity associated with glutamate release during tissue slicing. Immediately after slicing, slices were hemisected, trimmed, and placed in a holding chamber containing oxygenated ACSF_KA_ at room temperature for 1 h. Slices were then transferred to oxygenated regular ACSF containing (in mM): NaCl (130), NaHCO3 (30), KCl (3.50), KH2PO4 (1.10), MgCl2 (1.30), CaCl2 (2.50), and glucose (10). Slices were kept in the regular ACSF at room temperature for at least 30 min before recording. For recording, individual slices were transferred to a Warner Series 20 recording chamber (0.5 ml volume) mounted on the fixed stage of a Leica DM-LFS microscope (Leica Microsystems). The slices were maintained fully submerged and continuously perfused with ACSF heated to 32°C, and gassed with a 95–5% oxygen/carbon dioxide mixture. Neurons were visually identified using differential interference contrast (DIC) optics, infrared (IR) illumination, and an IR sensitive CCD camera (Orca ER, Hamamatsu, Tokyo Japan).

Standard whole-cell recordings in current- and voltage-clamp mode were obtained from BNST_ALG_ and PVN neurons using a MultiClamp 700B amplifier (Molecular Devices, Sunnyvale, CA), with a Digidata 1320A A-D interface, and pClamp 10 software (Molecular Devices). Current-clamp signals were filtered at 5 kHz and digitized at 10–20 KHz. Patch pipettes were fabricated from borosilicate glass (resistance 4–7 MΩ) and filled with a recording solution of the following composition (in mM): 130 K-gluconate, 2 KCl, 10 HEPES, 3 MgCl_2_, and 5 phosphocreatine, 2 K-ATP, 0.2 NaGTP.

For all experiments, whole-cell patch-clamp configuration was established only when the seal resistance was >1.5 GΩ. Neurons were excluded from analysis if they showed a resting membrane potential (*V*m) more positive than −50 mV and/or had an action potential that did not overshoot +10 mV. Series resistance (Rs) was bridge balanced and neurons with Rs > 25 MΩ were not included in analysis. Standardized protocols were used to determine the membrane properties of PVN and BNST neurons as previously described (Hammack et al., 2007). Briefly, the voltage response of neurons was determined using transient (750 ms) outward and inward current steps of 10–50 pA based on membrane input resistance (Rm) of neurons, with the maximum hyperpolarizing voltage deflection restricted to ~ −90 mV. Action potential properties were determined using an inward ramp of current (250 ms) sufficient to drive a single action potential. All electrophysiological data were analyzed with a custom made MATLAB 2009a script (Mathwork, Natick, MA). In a subpopulation of PVN neurons, 0.3% biocytin was included in the patch recording solution for morphological reconstructions as previously described (Hazra et al., 2011). PVNpc and PVNmc cells were distinguished from one another based on their relative medial / lateral location in the PVN, as well as by the apparent size of their soma (PVNpc = 10.7 ± 0.5 μm, *n* = 18; PVNmc = 18.8 ± 0.8 μm, *n* = 18).

### Statistical analysis

Statistical analyses were carried out using Prism 4 (GraphPad, La Jolla, CA). Tests for significant effect of cell type were performed using one way analysis of variance (ANOVA). To perform pair-wise comparisons following significant main effects in ANOVA, Bonferroni multiple comparisons test were used. A *t*-test was used when only two cell types (PVNpc and PVNmc) were being compared. For all tests, significance was defined at α = 0.05.

## Results

### Dual-immunofluorescence

#### GAD67 and VGLUT2 expression in the PVN

To determine the predominant amino-acid neurotransmitter phenotype of CRF neurons in the PVN and BNST_ALG_, we performed dual-immunofluorescence for CRF together with specific markers for glutamatergic and GABAergic neurons. We chose GAD67 as the marker of GABAergic neurons because previous studies had shown a high levels of GAD67 mRNA expression in the BNST (Pompolo et al., 2002). Similarly, although three different isoforms of VGLUT are found in the CNS (Ziegler et al., 2002; Herzog et al., 2004), VGLUT2 is the predominant glutamate transporter found in the PVN (Hrabovszky et al., 2005). Hence, we conducted dual-immunofluorescence studies to examine the relative degree of co-expression of CRF with GAD67 or VGLUT2 in the PVN and the BNST_ALG_.

**Figure 1:**
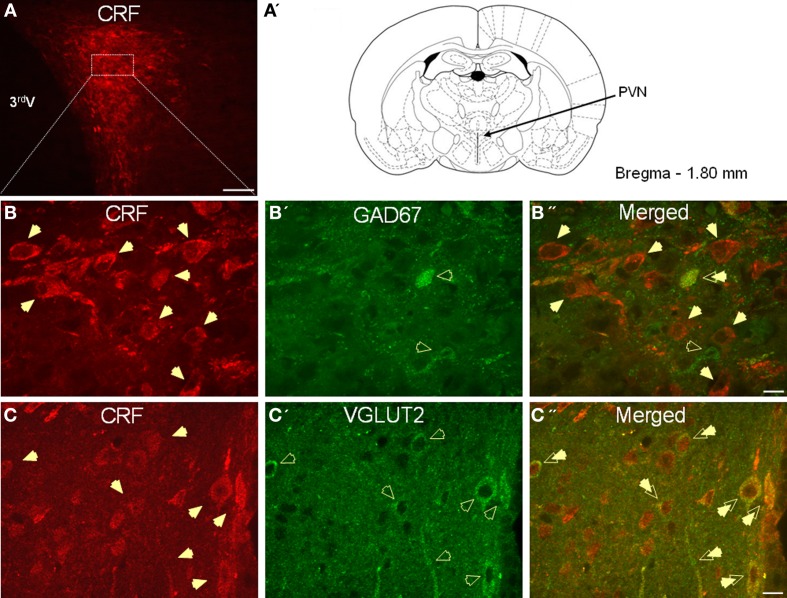
**(A)** Photomicrograph showing high somatodendritic immunoreactivity of CRF in the parvocellular neurons of the PVN (magnification 10×, scale bar 10 μ, 3rd V- third ventricle). **(B–B”):** Photomicrographs showing non-overlapping somatodendritic immunoreactivity of CRF (**B**, red, closed arrows) and GAD67 (**B'**, green, open arrows) in the PVN (**B”**, merged). Occasionally, CRF-positive cells demonstrate immunolabeling for GAD67 as indicated by double arrow. **(C–C”)** In contrast, CRF-immunoreactive neurons (**B**, red) are highly co-localized with VGLUT2-positive neurons (**B'**, green) in the parvocellular PVN (merged arrows, magnification 63×, scale bar 10 μm).

As expected, CRF-immunoreactivity in the PVN was mainly localized in a subpopulation of parvocellular neurons in the medial parvocellular division and, to a lesser extent, in a subpopulation of magnocellular neurons (Figures 1A–C). In contrast, GAD67-immunoreactivity showed high somatodendritic expression in the perinuclear division of the PVN and low expression in either the parvocellular or magnocellular subdivisions (Figure 1B'). Dual-immunofluorescence experiments demonstrated that CRF- and GAD67-immunoreactive neurons exist mainly in adjacent, but mutually exclusive, neuronal subpopulations in the hypothalamus, and only 13% (16/124) of CRF neurons in the PVN co-localize GAD67 (Figure 1B”). Conversely, VGLUT2-immunoreactivity showed high somatodendritic labeling throughout the PVN (Figure 1C') and our dual-immunofluorescence experiments revealed that the great majority (82%, 67/82) of CRF-positive neurons in the PVN also co-localized VGLUT2 (Figure 1C”). However, numerous VGLUT2 positive neurons were also observed in the PVN that did not co-localize CRF.

#### GAD67 and VGLUT2 expression in the BNST_ALG_

**Figure 2:**
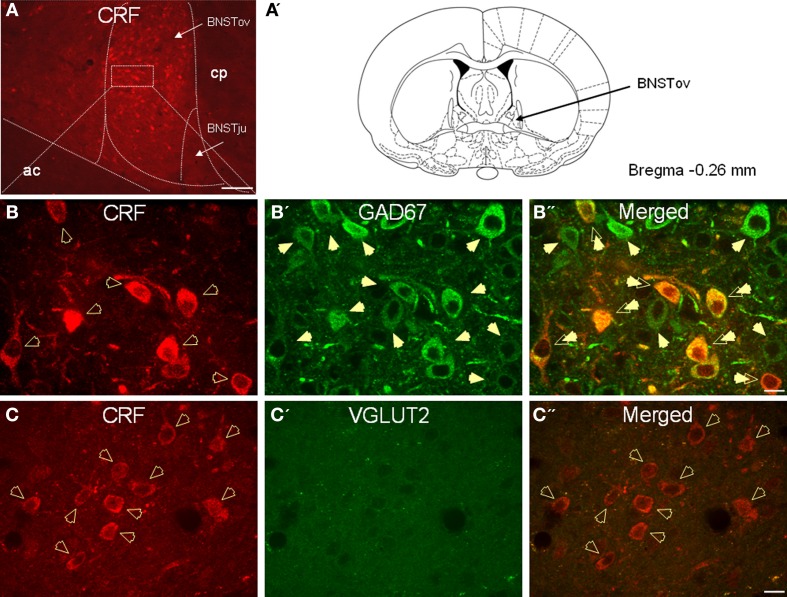
**(A)** Photomicrograph showing high somatodendritic immunoreactivity of CRF in the BNST_ALG_ (magnification 10×, scale bar 100 μ, ac, anterior commissure; cp, caudate putamen; BNSTov, oval nucleus of the BNST; BNSTju, juxtacapsular nucleus of the BNST). **(B–B”):** Photomicrographs showing high-level co-localization of CRF (**B**, red) and GAD67 (**B'**, green) in the BNST_ALG_ (**B”**, merged, double arrows). Virtually all CRF-immunoreactive neurons co-express GAD67, but numerous GAD67-positive neurons do not co-localize CRF in the BNST_ALG_. **(C–C”)** In contrast, CRF-immunoreactive neurons (**B**, red, open arrows) do not co-localize VGLUT2 (**C'**, green) in the BNST_ALG_ (magnification 63×, scale bar 10 μm).

In agreement with previous studies, CRF-immunoreactive neurons were concentrated in the oval and fusiform nuclei of the BNST, which also showed a dense network of CRF-positive fibers (Figures 2A–C). Consistent with previous *in situ* studies (Day et al., 1999; Pompolo et al., 2002), a high level of GAD67 somatodendritic immunoreactivity was observed throughout the BNST_ALG_ (Figure 2B'), and dual-immunofluorescence experiments further revealed that 95% (61/64) of CRF-positive neurons in the oval nucleus co-localized GAD67 (Figure 2B”). However, the majority of GAD67-positive neurons in the BNST_ALG_ do not co-localize CRF. Unlike GAD67, VGLUT2 immunoreactivity in the oval nucleus of the BNST_ALG_ was low, and restricted mainly to the neuropil where it presented as more punctuate-like labeling rather than the somatodendritic labeling of GAD67 (Figure 2C'). Furthermore, unlike GAD67, a somatic co-expression of VGLUT2 in CRF-positive neurons of the BNST_ALG_ was never observed (Figure 2C”).

Having determined that CRF neurons of the oval nucleus of the BNST_ALG_ and PVN could be differentiated based on their amino acid neurotransmitter phenotype, we were interested to see if they could be further differentiated based on their relative expression of other neuropeptides and their cognate receptors. Technical constraints limit the number of epitopes that can be labeled by antibodies in a single neuron and, hence, we next used a combination of whole-cell patch-clamp recording and single-cell RT-PCR to screen physiologically identified neurons in the parvocellular and magnocellular divisions of the PVN (PVNpc and PVNmc, respectively), and BNST_ALG_ for their expression of mRNA transcripts for multiple peptide neurotransmitters and their receptors.

### Single-cell RT-PCR

**Figure 3:**
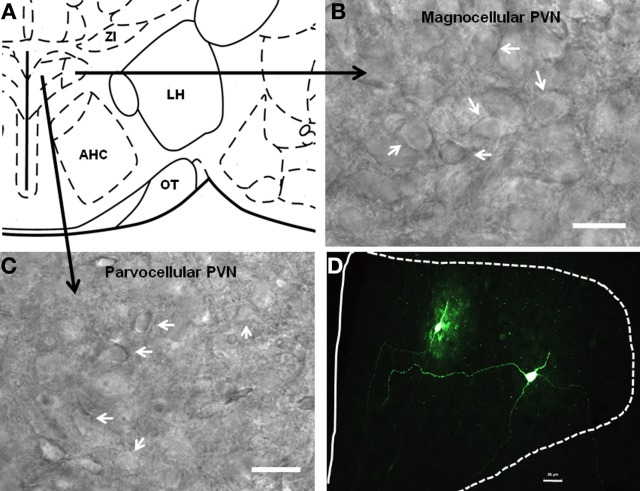
**Localization of parvocellular and magnocellular neurons in the PVN**. **(A)** A schematic diagram showing the recording sites from the PVN neurons. **(B,C)** Representative images showing the cytoarchitecture of magnocellular and parvocellular neurons under DIC illumination. **(D)** Immunofluoroscence image showing two anatomically reconstructed PVN neurons, one parvocellular and one magnocellular. Scale bar 25 μm.

Having visually identified putative PVNmc and PVNpc neurons, and BNST_ALG_ neurons, we first recorded their basic electrophysiological properties (see below) and then extracted cytosolic mRNA (see Methods). Figures 3A–C shows representative photomicrographs of putative magnocellular and parvocellular neurons in the PVN. In a subpopulation of neurons biocytin was included in the recording pipette to allow subsequent morphological reconstruction of the PVN neurons. Typical examples of a putative PVNmc (right) and PVNpc neuron (left) in the medial dorsal PVN are shown in Figure 3D. The combined results of our scRT-PCR analysis of mRNA transcripts obtain from neurons in the two divisions of the PVN and BNST_ALG_ are summarized below and in Tables 1, 3. The physiological properties of BNST_ALG_ and PVN neurons that express CRF transcripts are described briefly in the following section, and summarized in Table 2.

**Table 1:** **Transcriptomic analysis of CRF, non-CRF PVN cells, and Type I–III BNST_ALG_ neurons**.

**Neurons**	**CRF**	**CRFR1**	**CRFR2**	**OT**	**OTR**	**AVP**	**V1AR**	**V1BR**	**ENK**	**VGLUT2**	**GAD67**
**PVNpc (21**)											
CRF 13	13	0	7	10	0	0	0	10	1	12	0
Non-CRF 8	0	0	5	2	6	3	0	3	6	5	0
**PVNmc (22)**											
CRF 4	4	0	3	3	2	1	0	3	1	2	0
Non-CRF 18	0	1	15	17	3	2	0	4	10	18	0
**Type I BNST_ALG_ (20)**											
CRF 3	3	0	0	0	0	0	1	0	1	0	3
Non-CRF 17	0	4	0	0	0	0	16	1	1	0	17
**Type II BNST_ALG_ (20)**											
CRF 7	7	1	0	2	2	0	0	4	1	0	7
Non-CRF 13	0	1	1	1	6	0	0	7	9	0	13
**Type III BNST_ALG_ (20)**											
CRF 19	19	0	0	0	18	0	0	2	4	0	19
Non-CRF 1	0	1	1	0	1	0	0	1	1	0	1

**Table 2:** **Distinct membrane properties of CRF neurons in the PVN and BNST_ALG_**.

**CRF neurons**	**RMP (mV)**	**1st ISI (ms)**	**Rin (mΩ)**	**Spike**					
				**Amplitude (mV)**	**Half width (ms)**	**Threshold (mV)**	**Rise time (ms)**	**Decay time (ms)**	**fAHP (mV)**
PVNpc (12)	−56.3 ± 0.94	24.1 ± 3.2	1180 ± 102	70.1 ± 2.4	1.003 ± 0.072	−33.1 ± 0.89	0.36 ± 0.022	0.98 ± 0.094	−14.5 ± 0.9
PVNmc (4)	−55.2 ± 0.63	19.0 ± 3.3	373 ± 114^**^	73.3 ± 4.5	1.01 ± 0.042	−33.2 ± 1.12	0.37 ± 0.024	0.89 ± 0.030	−19.2 ± 2.6^*^
Type III BNST (19)	−66.4 ± 0.97^**^,^##^	26.6 ± 4.5	226 ± 17^**^	76.9 ± 1.4	1.04 ± 0.024	−39.0 ± 0.75^*^,^#^	0.44 ± 0.016	1.12 ± 0.045	−4.9 ± 0.5^**^,^##^

*,** p < 0.05 and 0.01 respectively, vs. PVNpc;

#,## p < 0.05 and 0.01 respectively, vs. PVNmc. ISI, inter-spike-interval; PVNpc, parvocellular neurons of the PVN; PVNmc, magnocellular neurons of the PVN.

#### Expression of mRNA transcripts in PVN neurons

Single-cell RT-PCR analysis was performed on mRNA isolated from 43 PVN neurons, of which 21 were putative PVNpc neurons, and 22 were putative PVNmc neurons. Previously, we have briefly described the genetic phenotype of a sample population of magnocellular PVN neurons (Dabrowska et al., 2011). Here, we extend these studies to include a more detailed examination of the phenotype of PVNpc and PVNmc neurons.

To verify the results from our immunohistochemical studies, all neurons were initially screened for the presence of mRNA transcripts for GAD67, as well as VGLUT1, VGLUT2, and VGLUT3. As expected, 86% (37/43 neurons) of PVN neurons screened in this study expressed mRNA transcripts for VGLUT2, and none expressed GAD67 transcripts, further confirming that PVN neurons have a predominantly glutamatergic phenotype. Moreover, consistent with results from previous *in situ* hybridization studies (Ziegler et al., 2002; Herzog et al., 2004; Singru et al., 2012), none of the PVN neurons screened expressed mRNA transcripts for either VGLUT1 or VGLUT3 (data not shown), suggesting that VGLUT2 is a unique identifier for glutamatergic neurons of the PVN. Having established that PVN neurons were predominantly glutamatergic we next examined the relative extent of co-expression of CRF mRNA transcripts with VGLUT2 transcripts. In 21 PVNpc neurons screened, 62% (13/21) expressed mRNA transcripts for CRF. Consistent with our immunohistochemical data, 92% (12/13) of PVNpc neurons that expressed CRF mRNA transcripts also expressed VGLUT2, and none expressed GAD67 transcripts.

Next, we examined the relative extent of co-expression of CRF transcripts in PVNpc neurons with transcripts for OT, AVP, and ENK, together with the cognate receptors for OT and AVP. As illustrated in Table 1, 77% of parvocellular CRF-positive neurons (10/13) were seen to co-express mRNA transcripts for OT and V1BR, 54% for CRFR2 (7/13), and only 8% expressed transcripts for ENK (1/13). None of the parvocellular CRF neurons examined in this study expressed transcripts for CRFR1, AVP, OTR, or V1AR mRNA. In contrast, 75% of non-CRF parvocellular neurons expressed ENK and OTR (6/8), 62% expressed VGLUT2 and CRFR2 (5/8), 37% expressed transcripts for AVP (3/8), and only 25% expressed transcripts for OT and V1BR (2/8).

We have previously briefly described the expression phenotype of a sample population of magnocellular CRF-positive neurons (Dabrowska et al., 2011). Here we extend these observations and compare their expression phenotype with that of parvocellular CRF neurons. Out of 22 PVNmc neurons screened in this study, only four expressed the mRNA for CRF. Like parvocellular CRF neurons, 75% of magnocellular CRF neurons (3/4) expressed mRNA transcripts for OT, CRFR2, and V1BR, 50% expressed transcripts for VGLUT2 (2/4), and 25% expressed transcripts for AVP and ENK (1/4). Interestingly, whereas no parvocellular CRF neurons expressed transcripts for OTR, 50% of magnocellular CRF neurons showed OTR expression. Finally, V1BR transcripts were preferentially expressed by CRF neurons and were never observed to co-express with AVP mRNA transcripts in either PVNpc or PVNmc, suggesting that V1BR does not serve as an autoreceptor in AVP neurons.

#### Expression of mRNA transcripts in BNST_ALG_ neurons

Previously, we have shown that three physiologically and genetically distinct subclasses of neurons are present in the BNST_ALG_, Type I–III (Hammack et al., 2007; Hazra et al., 2011). Type III BNST_ALG_ neurons are putative CRF neurons, since the majority (95%) of these neurons express mRNA transcripts for CRF, and display a unique ion channel expression pattern that correlates with their electrophysiological features (Hazra et al., 2011). To determine if CRF neurons of the BNST_ALG_ share a phenotype with parvocellular CRF neurons of the PVN we screened Type I–III BNST neurons for the presence of GAD67 and VGLUT1-3 transcripts. Significantly, all BNST_ALG_ neurons tested, irrespective of cell type, expressed GAD67 mRNA transcripts, but none of these neurons expressed transcripts for VGLUT1-3. These findings confirmed our immunohistochemical observations that CRF neurons in the BNST_ALG_ have a predominantly GABAergic phenotype.

Having established that CRF neurons of the PVN and BNST_ALG_ could be differentiated based on their amino-acid phenotype, we next examined whether additional differences could be found in their peptidergic phenotype. The results of this study are summarized in Table 1. Significantly, unlike parvocellular CRF neurons that showed 83% co-expression with OT mRNA transcripts, none of the Type III CRF neurons tested co-expressed OT. Conversely, whereas 95% of Type III neurons expressed transcripts for OTR (18/19), no parvocellular CRF neurons expressed OTR transcripts. Moreover, although 54% of parvocellular CRF neurons expressed CRFR2 transcripts, no expression was observed in Type III CRF neurons and, finally only 10% of Type III CRF neurons expressed V1BR transcripts (2/19), whereas 77% of the parvocellular CRF neurons did. Hence, CRF neurons in the BNST_ALG_ and the PVNpc would be expected to respond in distinctly different ways to local release of peptide neurotransmitters.

Examination of mRNA transcript expression for the different peptides and their receptors in Type I and Type II BNST_ALG_ neurons revealed that like CRF neurons, Type II BNST_ALG_ neurons never express V1AR transcripts, whereas 94% of non-CRF Type I BNST neurons do. Interestingly, 36% of those Type II neurons that co-expressed V1BR also expressed transcripts for CRF, similar to Type III neurons.

### Electrophysiological properties of putative CRF neurons in the PVN and BNST_ALG_

**Figure 4:**
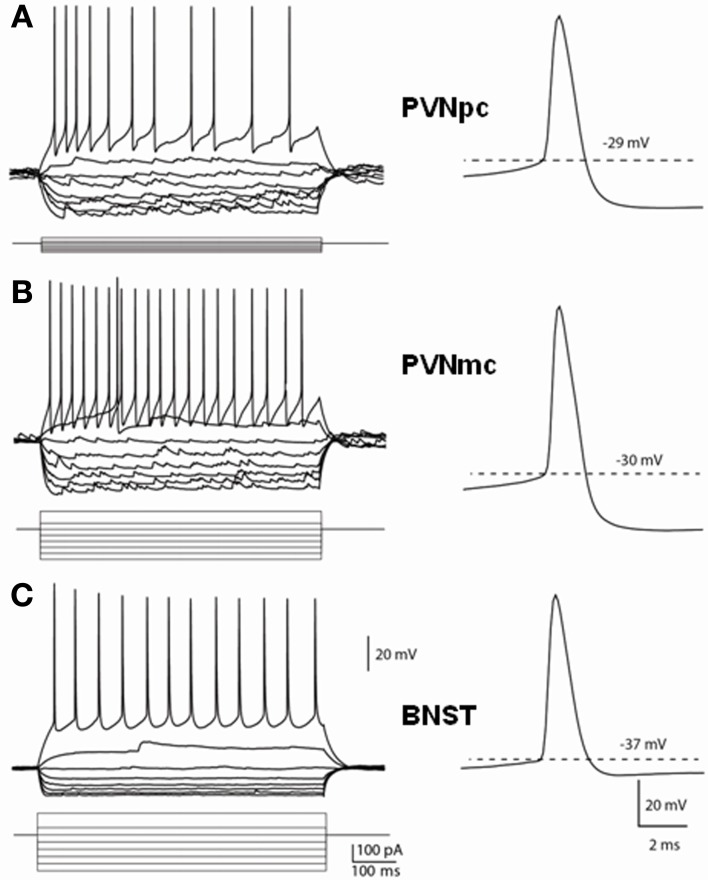
**Distinct membrane properties of putative CRF neurons in the PVN and type III CRF neurons in the BNST_ALG_**. Representative traces demonstrate the membrane responses of these neurons to membrane current injections and typical spikes. **(A)** PVNpc neurons have extremely high input resistance and high threshold for spike firing. When hyperpolarized, these neurons exhibited an inward rectification current. No outward rectification was observed during depolarization current injections. Right trace shows a typical spike that has high firing threshold and a prominent fAHP. **(B)** PVNmc neurons have smaller input resistance than that of PNVpc neurons and also have high threshold for spike firing. Single spike trace also shows this neuron has a high spike threshold and prominent fAHP. **(C)** In comparison to the PVN CRF neurons, CRF neurons in the BNST have lower input resistances, lower spike threshold and smaller fAHP. PVNmc, PVNpc represent magnocellular and parvocellular neurons of PVN respectively.

Having determined that CRF neurons of the PVN and BNST_ALG_ have significantly different neurochemical phenotypes, we then examined whether the electrophysiological properties of these neurons was also different. The typical voltage response of CRF neurons in the PVNpc, PVNmc, and BNST_ALG_ to transient (750 ms) depolarizing and hyperpolarizing current injection is illustrated in Figure 4. As can be seen in Figures 4A,B (left upper traces), CRF neurons from both PVN regions displayed a similar voltage response to transient current injection. Hence, all neurons recorded from each region showed a moderate level of spike-frequency adaptation in response to 750 ms depolarizing current injections (PVNpc *n* = 12, PVNmc *n* = 4). In contrast, BNST_ALG_ CRF neurons exhibited a mixed firing pattern in response to depolarizing current injection. Like CRF neurons in the PVN, 11/19 BNST_ALG_ CRF neurons showed spike-frequency adaptation in the first 175 ± 20 ms, and then settled down to a regular firing pattern for the remainder of the pulse. The remaining 8/19 neurons showed no spike-frequency adaptation and fired regularly throughout the current injection protocol (Figure 4C). In response to hyperpolarizing current injection, voltage transients of neurons in each region showed a marked time-independent anomalous rectification. However, on closer examination one-way ANOVA tests found significant differences in resting membrane potential [RMP, *F*_(2, 32)_ = 31.7, *p* < 0.001], input resistance [Rin, *F*_(2, 32)_ = 76, *p* < 0.001], spike threshold [*F*_(2, 32)_ = 13.7, *p* < 0.001], and fast afterhyperpolarization [fAHP, *F*_(2, 32)_ = 31.7, *p* < 0.001], as summarized in Table 2. *Post-hoc* Bonferroni tests revealed that parvocellular CRF neurons had a significantly higher mean Rin than both the magnocellular CRF neurons, and BNST CRF neurons. On the other hand, both the parvo- and magnocelluar CRF neurons in the PVN exhibited a more pronounced fast after-hyperpolarizing potential (fAHP) following each spike, as well as a more depolarized resting membrane potential compared to that of BNST_ALG_ CRF neurons. Additionally, CRF neurons in the BNST_ALG_ had a significantly more hyperpolarized threshold for action potential than both PVNpc and PVNmc neurons. The basic membrane properties of putative CRF neurons in the PVN and BNST_ALG_ are summarized in Table 2.

Previous studies in the PVN had reported that expression of the low-threshold calcium current, I_T_, was a unique identifier of parvocellular PVN neurons, which was markedly attenuated, or absent, in PVNmc neurons (Tasker and Dudek, 1991; Luther and Tasker, 2000), and that expression of this current may underlie differences in the firing patterns observed in these two regions. Similarly, we have shown that the firing pattern of BNST_ALG_ neurons is dependent on and interplay between I_T_ and the transient outward potassium current, I_A_ (Hammack et al., 2007). Hence, activation of I_T_ may play a significant role in regulating the firing activity in CRF neurons in both the PVN and the BNST_ALG_. As illustrated in Figure 4, CRF neurons in both the PVNpc and PVNmc failed to exhibit any of the rebound burst firing properties that were previously reported for type II neurons in the PVNpc (Luther and Tasker, 2000). However, burst firing was observed in a small population of non-CRF PVNpc neurons (data not shown). Alternatively, we reasoned that I_T_ activation may influence the firing activity of PVNpc CRF neurons by regulating the inter-spike interval (ISI) between the first two spikes of a train of action potentials. Indeed, when looking at all of the PVNpc and PVNmc neurons, including those that do not express CRF, PVNpc neurons displayed a tendency toward a shorter 1st ISI in comparison to PVNmc neurons (26.8 ± 4.7 vs. 42.5 ± 11.2 ms; *p* > 0.05), which may be indicative of more I_T_ activation. However, no significant difference was observed for the 1st ISI in CRF PVNpc and PVNmc neurons (24.1 ± 3.2 vs. 19.0 ± 3.3 ms, respectively), suggesting that CRF neurons in these two regions may share common properties that are distinct from non-CRF neurons. Although CRF neurons of the PVN and BNST_ALG_ share many basic electrophysiological properties; Table 2 illustrates that BNST_ALG_ CRF neurons can be differentiated from their PVN counterparts in some of their basic membrane properties, which could significantly influence their response to excitatory afferent inputs. These data are consistent with our dual-immunofluorescence results, further suggesting that CRF neurons in the PVN and BNST represent distinct neuronal populations.

### Expression of I_T_ channel subunits in CRF neurons of the PVN and BNST_ALG_

To determine if the differences observed in the 1st ISI may be dependent on differential expression of I_T_ channel subunits, we next examined the relative expression of mRNA transcripts for the I_T_ channel subunits Cav_3.1_, Cav_3.2_, and Cav_3.3_ in CRF neurons of the PVN and BNST_ALG_. As illustrated in Table 3, 54% (7/13) of CRF-positive neurons in the PVNpc expressed mRNA transcripts for the Cav_3.1_ subunit, whereas only 38% (5/13) expressed transcripts for the Cav_3.2_ subunit. In contrast, the only I_T_ channel subunit transcript expressed by PVNmc CRF neurons was the Cav_3.2_ subunit. Hence, 75% (3/4) of PVNmc CRF neurons co-expressed Cav_3.2_ subunit transcripts. Significantly, the majority of non-CRF PVNmc neurons (15/22) failed to show mRNA transcript expression for any of the I_T_ channel subunits (data not shown). In contrast to CRF neurons in the PVN, 85% of Type III CRF neurons in the BNST_ALG_ expressed mRNA transcripts for the Cav3.3 subunit, but did not express transcripts for either Cav3.1 or Cav3.2 subunits.

**Table 3:** **Comparison of mRNA transcript expression of I_T_ current channel subunits in PVN and BNST_ALG_ CRF neurons**.

**CRF neurons**	**Cav3.1**	**Cav3.2**	**Cav3.3**
PVNpc (13)	10	6	0
PVNmc (4)	0	3	0
Type III BNST _ALG_ (19)	0	0	16

## Discussion

In this study we have demonstrated that CRF neurons in the PVNpc and in the BNST_ALG_ display unique and regionally distinct expression patterns not only for the major amino acid neurotransmitters glutamate and GABA, but also for other neuropeptides and their receptors that are known to play a major role in the behavioral response to stress. Hence, our dual-immunofluorescence and scRT-PCR experiments revealed that PVNpc CRF neurons are predominantly glutamatergic, have the potential to synthesize and release oxytocin (OT), are unlikely to respond to local OT release, but could respond to local AVP and CRF release through activation of V1B and CRF2 receptors, respectively. Notably, PVNmc CRF neurons have a similar profile to that of PVNpc neurons. Conversely, Type III CRF neurons in the BNST_ALG_ are predominantly GABAergic, do not have the ability to release OT or AVP, but could respond to local OT and AVP release via activation of OT and V1B receptors. Moreover, CRF neurons in the BNST_ALG_ do not express CRF2 receptors and, hence, local CRF release would be predicted to differentially modulate the activity of CRF neurons in these two regions. Finally, our physiological data further suggests that excitatory afferents would be more likely to drive PVNpc CRF neurons to fire action potentials than they would CRF neurons of the BNST_ALG_, even though the action potential threshold is lower in BNST_ALG_ neurons, due to PVNpc neurons having a more depolarized resting membrane potential and higher input resistance.

Vesicular glutamate transporters are the main regulators of glutamate uptake into synaptic vesicles prior to its release from axon terminals (Fujiyama et al., 2001). To date three isoforms have been identified, VGLUT1-3, each of which show differential expression in the CNS (Ziegler et al., 2002; Herzog et al., 2004). Here we report that CRF neurons of the PVNpc and PVNmc exclusively express mRNA transcripts for VGLUT2, but not VGLUT1 or VGLUT3. The data is consistent with previous *in situ* hybridization and immunocytochemical studies showing that VGLUT2 is the predominant isoform expressed in the PVN (Ziegler et al., 2002), and that hypophysiotropic parvocellular CRF neurons of the PVN express VGLUT2 (Lin et al., 2003; Hrabovszky et al., 2005). Similarly, VGLUT2 has been reported to be the predominant glutamate transporter expressed by almost all hypothalamic neuroendocrine neurons, including CRF, thyrotropin releasing hormone (TRH; Herman et al., 2002; Hrabovszky and Liposits, 2008), OT, and AVP expressing neurons (Takamori et al., 2000; Herzog et al., 2001; Dabrowska et al., 2011), suggesting that glutamate may be the principal neurotransmitter released by these neurons under basal firing conditions. Unlike fast amino-acid synaptic transmission, release of peptide neurotransmitters is slow, requiring high frequency firing to elicit release, and is thought to modulate subsequent fast amino-acid transmission (Kits and Mansvelder, 2000; Greengard, 2001). Hence, transmission in efferent pathways from PVN CRF neurons may switch from solely glutamate, to glutamate and CRF during times of high PVN activation. Significantly, CRF has been shown to facilitate glutamatergic transmission in multiple brain regions (Liu et al., 2004; Rainnie et al., 2004; Hahn et al., 2009) but see (Gallagher et al., 2008), suggesting that concurrent activation of postsynaptic glutamate and CRF receptors may enhance HPA axis activity in response to chronic or intense stress stimuli.

Consistent with previous immunohistochemical studies (Cullinan et al., 2008) our scRT-PCR data confirm that neurons in the PVNmc (Dabrowska et al., 2011) and PVNpc do not express GAD67 mRNA transcripts. However, we have observed GAD67-immunoreactive neurons at high levels in the perinuclear region adjacent to the PVN, which might be consistent with the presence of local inhibitory interneurons reported in earlier *in situ* hybridization studies of GAD67 expression in the PVN (Cole and Sawchenko, 2002). Indeed, CRF neurons of the medial PVNpc were shown to be targets of GABAergic inputs originating from neurons in the perinuclear zone (Roland and Sawchenko, 1993; Boudaba et al., 1996; Miklos and Kovacs, 2002), as well as from extrinsic sources such as the BNST (Herman and Cullinan, 1997).

The BNST is thought to be a major relay site for limbic input into the PVN (Cullinan et al., 1993; Dong et al., 2001; Zhu et al., 2001; Crane et al., 2003) and, notably, the PVNpc receives the heaviest BNST projection from the oval and fusiform nuclei, which contain the CRF neurons (Dong et al., 2001; Dong and Swanson, 2006). Lesion studies have shown that the anterior BNST mediates HPA axis activation, while the posterior BNST is involved in HPA axis inhibition (Choi et al., 2007) and therefore it has been suggested that CRF neurons of the anterior BNST might co-express glutamate (Silverman et al., 1989). However, we have shown that CRF neurons of the BNST_ALG_ do not express mRNA transcripts for any of the three VGLUT isoforms and, instead, express transcripts and protein for GAD67. Our results are consistent with previous *in situ* hybridization studies showing that the BNST is predominantly a GABAergic structure (Sun and Cassell, 1993; Bowers et al., 1998; Day et al., 1999; Bali et al., 2005). At first glance these results would seem to contradict the results of the lesion studies showing activation of the HPA axis by the anterior BNST. However, Choi and colleagues proposed that it was the anterior BNST, ventral to the commissure, in the region of the fusiform nucleus that was responsible for activation of the PVN, and not the BNST_ALG_ (Choi et al., 2007). The oval nucleus of the BNST_ALG_ projects heavily to the fusiform nucleus (Dong et al., 2001) and, hence, inhibition of fusiform neurons may functionally disinhibit the PVN.

More recently, it has been proposed that neurons in the region of the fusiform nucleus are glutamatergic (Georges and Aston-Jones, 2002; Jennings et al., 2013). However, there is some question about the validity this observation as the results from several *in situ* hybridization studies, including those reported in the Allen Brain Atlas, suggest that only a few putative VGLUT2-3 mRNA expressing neurons are present in this region (Herzog et al., 2001; Kudo et al., 2012). Nevertheless, if CRF neurons of the BNST_ALG_ are involved in the acute response to stressors via an indirect pathway then inhibition of putative glutamatergic neurons in the ventral BNST would paradoxically decrease activation of the PVN. An alternative mechanism might be that activation of GABA/CRF neurons in the BNST_ALG_ could indirectly activate the HPA axis by dis-inhibiting GABAergic interneurons of the perinuclear zone. However, no study to date has reported direct projections from the BNST_ALG_ to this region.

Notably, Day and colleagues have suggested that systemic stressors, such as infection, do not activate CRF neurons in the BNST_ALG_, but instead activate ENK neurons (Day et al., 1999) as measured by alterations in c-*fos* expression. However, Cullinan and colleagues (Cullinan et al., 1995) have reported that psychological stressors, such as restraint stress, cause a dramatic increase in the expression of another immediate early gene, Zif/268, but caused only low level c-*fos* expression. Hence, activation of CRF neurons on the BNST_ALG_ may be critically dependent on the nature of the stressor, and detection of their activation is dependent on which immediate early gene is screened.

Interestingly, the majority of PVN CRF neurons also co-expressed mRNA transcripts for OT, and some of the magnocellular neurons also co-expressed transcripts for the OTR. OT is classically viewed as an anxiolytic neurotransmitter (Mccarthy et al., 1996; Ebner et al., 2005; Lee et al., 2009) and, hence, the potential for OT expression in parvocellular CRF neurons is intriguing and raises the possibility that these neurons could switch their neurochemical phenotype depending on environmental demands. Dynamic regulation of neuronal phenotype is not without precedence in the CNS. Hence, during lactation tuberoinfundibular dopamine neurons begin to express ENK, which prevents dopamine from inhibiting prolactin secretion (Merchenthaler, 1993). Dynamic changes in the expression of both CRF1 and CRF2 receptors were also reported in hypothalamic magnocellular neurons expressing AVP and OT in response to hyperosmotic stress (Arima and Aguilera, 2000). It is possible that prolonged periods of stress may dynamically regulate the neurochemical phenotype of parvocellular CRF neurons in a similar manner.

Moreover, our data showing co-expression of OT and OTR transcripts is consistent with previous autoradiography studies showing OTR expression in OT neurons (Adan et al., 1995), suggesting that the OTR in this cell population might serve as an autoreceptor to regulate OT release (Freund-Mercier and Richard, 1984). Interestingly, we reported previously that the majority of CRF neurons in the BNST_ALG_ also expressed mRNA transcripts for the OTR (Dabrowska et al., 2011), suggesting that OT could regulate the activity of CRF neurons in the BNST_ALG_ and therefore directly modulate affective behavior. In the current study we show that the majority of non-CRF neurons in the PVN have the potential to produce OTR, suggesting that local OT release could differentially modulate the excitability of the parvocellular neurons, and therefore impact the activity of the HPA axis as it was suggested before (Neumann et al., 2000). However, mRNA transcript expression does not necessarily translate into expression of the mature peptide, since we have previously demonstrated relatively sparse co-localization of CRF- and OT-neurons in the PVN (Dabrowska et al., 2011).

It is noteworthy that we saw no co-expression of CRF with AVP in either the PVN or BNST_ALG_, particularly considering that CRF and AVP have previously been reported to co-localize in both the PVNpc and PVNmc (Ma et al., 1997; Arima et al., 2001). Sawchenko and colleagues have reported that acute stress can cause a delayed induction of AVP mRNA transcripts in PVNpc CRF neurons (Kovacs and Sawchenko, 1993; Sawchenko et al., 1993). It is possible that in our unstressed animals the expression level of AVP transcripts is just below our detection threshold. In addition, is has been suggested that two populations of CRF neurons exist in the PVNpc, one that co-express AVP and one that does not (Whitnall and Gainer, 1988). It is possible that with our relatively small sample size we have unintentionally biased our recordings for scRT-PCR toward the former population.

We have also shown that Type III CRF neurons in the BNST_ALG_ and PVNpc CRF neurons both express mRNA transcripts for V1BR, but not V1AR, whereas non-CRF Type I neurons of the BNST_ALG_ express V1AR but not V1BR. These findings are consistent with previous studies showing a high level of expression of the V1B receptor in the PVN, where it is known to play an key role in modulating activity of the HPA axis (Nair and Young, 2006); for review see (Roper et al., 2011). Significantly, V1B receptor knockout mice have been shown to have a blunted ACTH response to both acute and repeated stress, however, this response is critically dependent on the context of the stressor (Lolait et al., 2007). Our results showing V1BR transcript expression in BNST_ALG_, as well as PVN, CRF neurons would suggest that the effect of the receptor knockout would depend on whether or not a particular stressor activated one or both of these pathways. Consistent with this hypothesis recent studies with the non-peptide V1B receptor antagonist, suggest that extra-hypothalamic V1B receptors may play a critical role in the regulation of affect (Salome et al., 2006). We have preliminary scRT-PCR evidence suggesting that those Type I BNST_ALG_ neurons that express V1AR co-express GABA and either somatostatin or enkephalin (Hazra and Rainnie, unpublished observations). Hence, local AVP release might differentially regulate sub-populations of BNST_ALG_ neurons and modulate different behavioral outputs. Consistent with this notion, previous studies have suggested a complementary or cooperative effect of AVP receptor activation on behavioral output (Ring, 2005; Veenema and Neumann, 2008). Central V1B receptors were shown to mediate anxiety (Ishizuka et al., 2010), while V1A receptors in the BNST were positively correlated with maternal aggression (Bosch et al., 2010; Caughey et al., 2011). The BNST is known to mediate both anxiety-like and maternal aggression behavior, thus our results suggest that in the BNST, Type III CRF neurons and Type I neurons might mediate distinct anxiety- and aggression-related behavioral outcomes, respectively.

Finally, using *in vitro* patch-clamp techniques, we have demonstrated that CRF neurons of the PVN and BNST_ALG_ display similar, and yet distinct basic membrane properties. Previously, we have identified three electrophysiologically and genetically distinct cell types (Type I–III) in the BNST_ALG_ (Hammack et al., 2007; Hazra et al., 2011), and have further identified Type III neurons as corresponding to the CRF containing cell population (Martin et al., 2010; Dabrowska et al., 2011). Previous studies in the PVN have also identified three neuronal subtypes (Type I–III) based on their electrophysiological properties (Hoffman et al., 1991; Tasker and Dudek, 1991; Luther and Tasker, 2000), and type I and II neurons were subsequently identified as being magnocellular and parvocellular neurons, respectively (Luther and Tasker, 2000). Significantly, the parvocellular CRF neurons recorded in our study have many electrophysiological properties similar to those previously reported for the neurosecretory subpopulation of type II parvocellular PVN neurons (Tasker and Dudek, 1991); in that PVNpc CRF neurons displayed moderate spike-frequency adaptation of the action potential firing pattern from rest, and had a pronounced post-spike fast AHP. However, no rebound low-threshold burst firing activity was observed in PVNpc CRF neurons following transient hyperpolarizing current injection. Two distinct cell populations have been reported in the PVNpc (Hermes et al., 1996), one of which shows robust rebound low-threshold burst firing activity (type III) and one that does not (type II). Consistent with this observation we observed a subpopulation of non-CRF PVNpc neurons that did show low-threshold burst firing activity. Moreover, CRF PVNpc neurons showed a marked time-independent anomalous rectification of the voltage transient in response to hyperpolarizing current injection similar to that previously reported in type II and III PVNpc neurons.

Like the PVNpc CRF neurons, the PVNmc CRF neurons in this study also exhibited a marked time-independent anomalous rectification. However, PVNmc neurons have previously been reported to lack this time-independent inward rectification (Tasker and Dudek, 1991). Consistent with the previous studies, non-CRF PVNmc cells in the current study did not exhibit the inward rectification, indicating that the CRF PVNmc cells exhibit electrophysiological properties more similar to that of PVNpc neurons than other PVNmc neurons. Another distinguishing property of PVNmc, or Type I neurons, is a large A-type K^+^ current (I_A_; Luther and Tasker, 2000). Although we did not measure I_A_ directly in this study, we did observe an ~200 ms delay in the onset to the first action potential in 3/4 CRF PVNmc cells compared to PVNpc neurons in response to low intensity current injection (Figure 4B). However, due to the small sample size of PVNmc neurons (*n* = 4) it is impossible to draw any definitive conclusions about the factors mediating this response.

Intriguingly, CRF neurons of the PVN and BNST_ALG_ had many overlapping physiological properties such as a regular firing pattern, and a strong inward-rectification of the voltage response to hyperpolarizing current injection. However, consistent with differences observed in our immunohistochemical and transcriptomic analysis, Type III CRF neurons and PVNpc CRF neurons were significantly different in many other aspects of their membrane properties. Hence, PVNpc CRF neurons had a much higher input resistance (1180 MΩ) compared to the CRF neurons in the BNST_ALG_ (226 MΩ), which suggests that the same excitatory input would cause a greater depolarization in PVNpc CRF neurons. Although CRF neurons in the BNST_ALG_ had a more hyperpolarized threshold for action potential (−39 mV) than PVNpc CRF neurons (−33 mV), PVNpc CRF neurons also showed a more depolarized resting membrane potential (−56 mV) compared to BNST_ALG_ CRF neurons (−66 mV), which together with the higher input resistance suggests that excitatory synaptic input would be more likely to drive PVNpc CRF neurons to action potential threshold than those in the BNST_ALG_. Furthermore, previous studies have shown that PVNpc neurons exhibit a strong I_T_ current that contributes to a low-threshold spiking pattern and only a little I_A_ (Tasker and Dudek, 1991; Luther and Tasker, 2000). Although we did not observe burst firing in the current study, we have confirmed that the parvocellular PVN neurons, which expressed CRF, also co-expressed Cav_3.1_ and Cav_3.2_ subunits mediating the I_T_ currents. Conversely, the Type III putative CRF neurons in the BNST do not exhibit low-threshold firing indicative of an I_T_ current (Hammack et al., 2007), but do show significant I_A_ currents (Rainnie et al., 2013). However, in the current study we have shown that the great majority of Type III, putative CRF neurons of the BNST_ALG_ co-expressed Cav_3.3_ subunit. The lack of low-threshold firing in these neurons, despite the probable presence of voltage-gated calcium channels, is most likely due to the opposing I_A_ current found in these cells.

PVNpc CRF neurons are responsible for a rapid activation of the HPA axis to initiate the endocrine response to stress stimuli (Rivier and Vale, 1983), while the CRF neurons in the BNST_ALG_ are thought to modulate the affective component of the stress response, which is most likely context-dependent and slower (Walker et al., 2009) but see Sterrenburg et al. (2012). Thus, the functional separation of these two neuronal populations might be partially explained by their distinct membrane properties and concomitant ion channel expression pattern.

In conclusion, we reported that although CRF neurons in the PVN and the BNST_ALG_ share the same neuropeptide phenotype, they represent distinct neuronal populations. Hypothalamic CRF neurons are glutamatergic (excitatory), whereas the CRF neurons in the BNST_ALG_ are GABAergic (inhibitory). Functional separation of these two groups of CRF neurons can be further distinguished by their distinct membrane properties, such that hypothalamic CRF neurons are more likely to be rapidly activated in response to stress to initiate HPA axis activation, while CRF neurons in the BNST_ALG_ display membrane properties which suggest that greater stimulus is needed to reach action potential threshold in these neurons. Consistent with this observation, CRF neurons in the BNST are thought to modulate the affective component of the stress response, and previous studies have suggested that in contrast to the PVN neurons, CRF neurons in the BNST may only be activated in response to chronic, but not acute stress (Kim et al., 2006). Furthermore, we have shown that OT and AVP as well as their cognate receptors co-exist in intimate relationships with CRF neurons in both PVN and the BNST_ALG_. However, OT is more likely to regulate the excitability of CRF neurons in the BNST_ALG_ than in the PVN, therefore OT-CRF neurotransmission seems to be more likely involved in the affective component of the stress response than in the classic endocrine response to stress. In contrast, V1BR were widely expressed in both PVN and the BNST CRF neurons, which suggests that AVP may be involved in both HPA axis activation as well as in the affective component of the stress response, which is mediated by CRF neurons in the BNST_ALG_.

### Conflict of interest statement

The authors declare that the research was conducted in the absence of any commercial or financial relationships that could be construed as a potential conflict of interest.
